# Quercetin targets the Ccl4–Ccr5 axis to relieve neuropathic pain after spinal cord injury

**DOI:** 10.1063/5.0253463

**Published:** 2025-07-24

**Authors:** Xiangsheng Zhang, Yu Cao, Lu Li, Yike Liu, Pengyu Zhou, Yupei Lai, Suo Wang, Yuefen Zuo, Jiahao Chen, Chuying Chen, Jiurong Cheng, Yingdong Deng, Ziqiang Lin, Simin Tang, Peng Sun, Yan Zhang, Jun Zhou

**Affiliations:** 1 Department of Anesthesiology, The Third Affiliated Hospital, Southern Medical University, Guangzhou 510060, China; 2 Department of Anesthesiology, Shunde Hospital of Guangzhou University of Chinese Medicine, Foshan, China; 3 Department of Anesthesiology, Sun Yat-sen University Cancer Center, Guangzhou 510060, People's Republic of China and State Key Laboratory of Oncology in South China, Collaborative Innovation Center for Cancer Medicine, Guangzhou 510060, People's Republic of China; 4 Department of Pain, Union Hospital, Tongji Medical College, Huazhong University of Science and Technology, Wuhan 430000, China

## Abstract

Spinal cord injury (SCI) severely disrupts the central nervous system, with neuropathic pain (NP) emerging as a prevalent and challenging complication, affecting approximately two-thirds of affected individuals. This study aims to explore the immune landscape and potential drug therapeutic targets associated with NP post-SCI using single-cell and bulk RNA sequencing. We identified 1050 differentially expressed genes enriched in cytokine interactions and inflammatory pathways, including key pain-related genes like Itgb2, Ccr5, Fcrg3, and Adora3, through weighted gene co-expression network analysis and immune infiltration analysis. Cell communication analysis revealed the pivotal role of the Ccl4–Ccr5 signaling axis in the interaction between macrophages and natural killer cell, thereby intensifying neuroinflammatory responses and aberrant nociceptive signaling, which may contribute to apoptosis after SCI. Molecular docking and molecular dynamics simulations showed that quercetin had stable binding with Ccr5 and identified potential amino acid binding sites TYR-108 and PHE-109. In vivo experiments demonstrated that Ccr5 inhibitors and quercetin effectively improved the Basso mouse scale and mechanical withdrawal threshold score, concurrently attenuating spinal tissue apoptosis. Therefore, we propose that quercetin and Ccr5 inhibitors could potentially treat NP post-SCI by inhibiting the Ccl4–Ccr5 pathway and reducing apoptosis, providing new treatment avenues.

## I. INTRODUCTION

Spinal cord injury (SCI) is a debilitating condition of the central nervous system caused by trauma, disease, or degeneration.^1,2^ With high incidence and disability rates, SCI has emerged as a major global public health concern. Beyond the loss of motor and sensory functions, SCI leads to complex complications, notably neuropathic pain (NP).^3,4^ NP is a chronic pain condition arising from lesions or diseases affecting the somatosensory system,^5^ which is particularly prevalent and challenging to treat. Epidemiological data indicate that 72% of patients experience persistent symptoms at 6 months post-injury, with 69% still experiencing persistent pain at 12 months.^6^ Notably, a study by Sommer *et al.*^7^ revealed that sustained activation of reactive astrocytes, microglia, infiltrating macrophages, and abnormal neuronal activity post-SCI is closely related to NP occurrence. Unlike other forms of NP, such as HIV neuropathy, individuals with SCI-associated NP often endure intense and excruciating pain while showing limited response to conventional pain medications, significantly compromising their overall quality of life.^8^ Therefore, in-depth research on NP following SCI to explore potential therapeutic targets is crucial for improving patient outcomes.

Studies show that the development of NP post-SCI is closely linked to changes in immune metabolism and inflammatory responses.^9–11^ To better understand the pathological mechanisms of NP post-SCI, this study aims to identify key genes, immune cellular components (CC), and potential downstream signaling pathways associated with NP by integrating single-cell and bulk transcriptome data. We identified 1050 differentially expressed genes (DEGs) significantly enriched in immune-related pathways, including cytokine–cytokine receptor interactions,^12^ T-cell differentiation,^13,14^ and the JAK–STAT signaling pathway.^15^ Single-sample gene set enrichment analysis (ssGSEA) revealed differences in immune infiltration levels between SCI and control samples, notably involving macrophages, T cells, and natural killer (NK) cells. Further analysis indicated that immune-related pain genes such as Itgb2, Adora3, Fcrg3, and Ccr5 significantly increase in spinal cord tissue post-SCI, playing crucial roles in NP development.

Subsequently, the cell expression pattern of the hub gene was identified from the scRNA-seq data. To gain insights into the specific roles these genes and immune cells play in NP after SCI, CellChat,^16^ and NicheNet^17^ analyses determined that the Ccl4–Ccr5 ligand–receptor pair is pivotal in mediating communication between macrophages and NK cells. This interaction potentially exacerbates pain transmission by amplifying neuroinflammation.^18,19^ Considering the biological effects of receptor cell signaling, NicheNet analysis identified a close association between the activation of the Ccl4–Ccr5 signaling pathway and increased apoptosis following SCI, shedding new light on the mechanisms of cell death in SCI. Apoptosis, initially recognized as a form of programmed cell death, has been notably linked to SCI and NP in previous studies.^20^ To further investigate potential therapeutic targets, molecular docking, and molecular dynamic simulation studies highlighted significant binding sites of quercetin with Ccr5 and Itgb2, suggesting beneficial therapeutic effects of quercetin, which were confirmed in subsequent experiments.^21,22^ Quercetin, a flavonoid known for its anti-inflammatory and antioxidant properties in treating neurological disorders, presents a promising avenue for intervention.^23,24^ Consequently, targeting the Ccl4–Ccr5 signaling pathway with quercetin or Ccr5 inhibitors may provide a novel therapeutic strategy for managing NP following SCI.

## II. RESULTS

### A. Preprocessing data and screening for DEGs and enrichment analysis

**FIG. 1.**
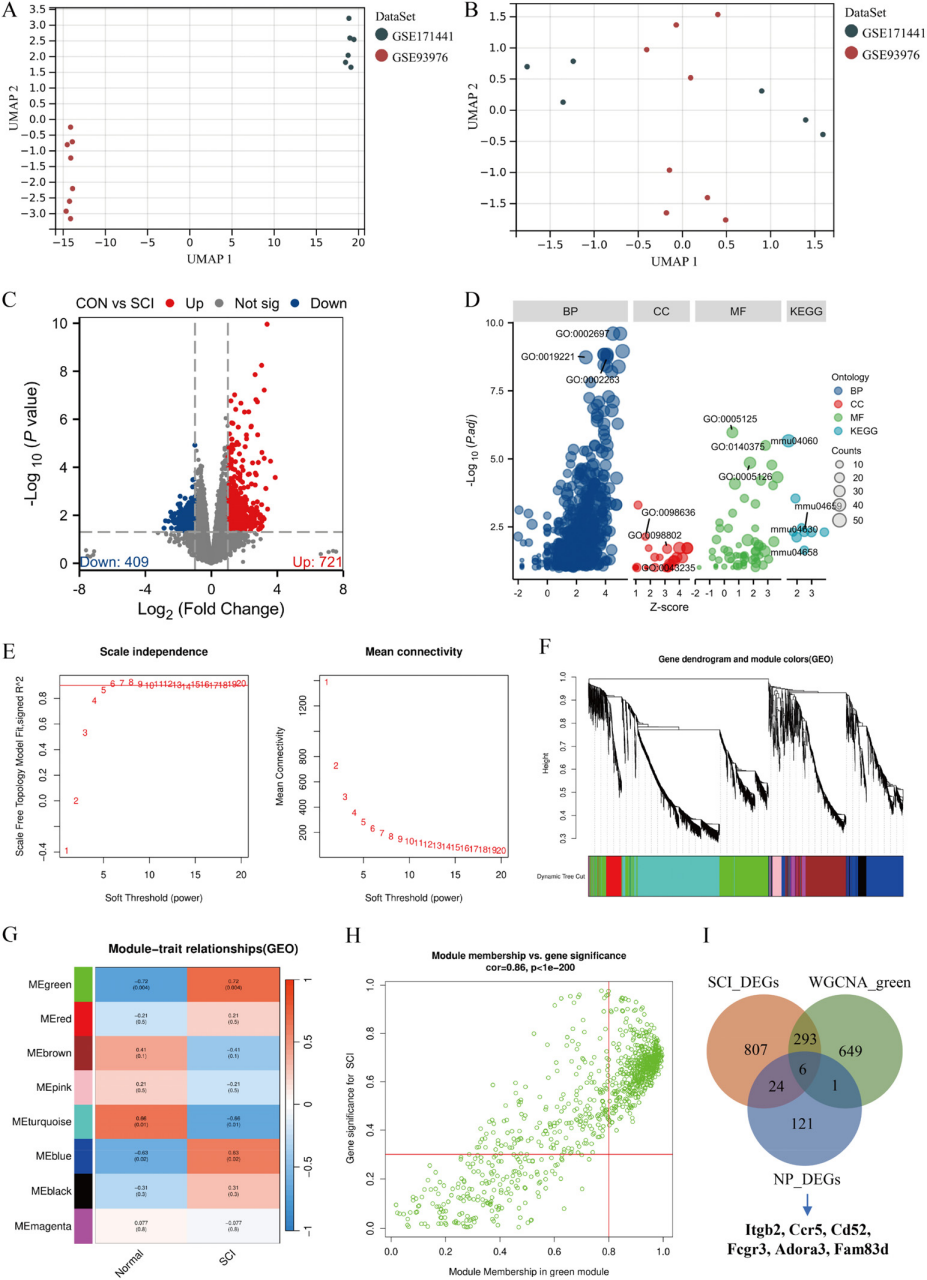
Identification of pain-related DEGs after spinal cord injury as hub genes. (a) UMAP plot showing the sample distribution of the SCI dataset before batch effect removal, with each point representing one sample. (b) Sample distribution for each dataset after batch effect removal. (c) Volcano plot of DEGs, where red represents significantly upregulated genes, blue represents significantly downregulated genes, and gray represents genes with no significant difference. (d) Bubble plot showing Gene Ontology (GO) and Kyoto Encyclopedia of Genes and Genomes (KEGG) analysis. The size of the circles indicates the number of DEGs enriched in each category (the larger the circle, the more DEGs enriched), and the colors represent different categories: biological process (BP), cell component (CC), molecular function (MF), and KEGG enrichment pathway. (e) β = 6 (scale-free R^2^ = 0.85) was chosen to construct a scale-free network. (f) A cluster tree and a co-representation network module are constructed. (g) Correlation between modules (columns) and clinic traits (rows). Red indicates a positive correlation, and blue indicates a negative correlation. Each cell contains a correlation coefficient and a p-value. (h) Scatter plots showing correlations between gene significance (GS >0.3) and module membership (MM >0.8). (i) The Venn diagram shows the intersection of genes between module genes, NP_DEGs, and SCI_DEGs.

Data downloaded from the Gene Expression Omnibus (GEO) database were organized and summarized in Table I. Initially, the GSE171441 and GSE93976 datasets were merged, resulting in six CON samples and eight SCI samples. Subsequently, batch effects were mitigated, and the dataset was normalized to confirm the reliability of the data [Figs. 1(a) and 1(b)].

**TABLE I.** The dataset involved in this study. Note: Download this file GSE111216_Parisien_Suppl_Table_04_gene_SC_CTRvsSNI.xlsx in GSE111216.

Datasets	Experiment type	Platform
GSE171441	Expression profiling by high-throughput sequencing	GPL16417 Illumina (*Mus musculus*)
GSE93976	Expression profiling by high-throughput sequencing	GPL13112 Illumina (*Mus musculus*)
GSE111216	Expression profiling by high-throughput sequencing	GPL13112 Affymetrix (*Mus musculus*)
GSE182803	Expression profiling by high-throughput sequencing	GPL24247 Illumina (*Mus musculus*)

A total of 1130 DEGs (721 upregulated and 409 downregulated genes) in the combined gene expression matrix between the control and SCI conditions were identified. These DEGs were depicted in the volcano plot shown in Fig. 1(c). The Gene Ontology (GO) and Kyoto Encyclopedia of Genes and Genomes (KEGG) functional analysis results are exhibited in Fig. 1(d), with visual IDs shown in Table II. GO analysis indicates that the DEGs primarily participate in three cellular functions. In biological processes (BP), significant changes were observed in the regulation of the immune effector process, cytokine-mediated signaling pathway, and cell activation involved in the immune response. Cellular component (CC) analysis primarily showed variations in the protein complex involved in cell adhesion and the plasma membrane signaling receptor complex. Molecular function (MF) analysis revealed changes in immune receptor activity and cytokine receptor binding. KEGG pathway analysis indicates significant DEG enrichment in several pathways, including cytokine–cytokine receptor interaction, Th17 cell differentiation, the JAK–STAT signaling pathway, and Th1 and Th2 cell differentiation.

**TABLE II.** ID of GO and KEGG enrichment results.

Ontology	ID	Description	GeneRatio	p_value	p.adjust	Count
BP	GO:0002697	Regulation of immune effector process	50/972	5.18 × 10^−14^	2.49 × 10^−10^	50
BP	GO:0019221	Cytokine-mediated signaling pathway	46/972	2.99 × 10^−12^	1.85 × 10^−9^	46
BP	GO:0002263	Cell activation involved in immune response	40/972	3.4 × 10^−12^	1.87 × 10^−9^	40
CC	GO:0098636	Protein complex involved in cell adhesion	9/984	3.14 × 10^−5^	0.0073	9
CC	GO:0098802	Plasma membrane signaling receptor complex	17/984	0.0003	0.0203	17
CC	GO:0043235	Receptor complex	27/984	0.0016	0.0564	27
MF	GO:0005125	Cytokine activity	29/965	1.25 × 10^−9^	1.07 × 10^−6^	29
MF	GO:0140375	Immune receptor activity	21/965	7.55 × 10^−9^	3.22 × 10^−6^	21
MF	GO:0005126	Cytokine receptor binding	32/965	4.92 × 10^−8^	1.4 × 10^−5^	32
KEGG	mmu04060	Cytokine–cytokine receptor interaction	41/476	7.24 × 10^−9^	2.17 × 10^−6^	41
KEGG	mmu04659	Th17 cell differentiation	16/476	0.0001	0.0050	16
KEGG	mmu04630	JAK–STAT signaling pathway	21/476	0.0002	0.0076	21
KEGG	mmu04658	Th1 and Th2 cell differentiation	13/476	0.0007	0.0226	13

### B. Identification of SCI-related NP genes and analysis of their immune microenvironment

Weighted gene co-expression network analysis (WGCNA) was applied to construct a scale-free network, with β = 6 (scale-free R^2^ = 0.85) [Fig. 1(e)]. After the setting displayed a similarity greater than 0.75, resulting in eight modules [Fig. 1(f)]. The correlation between each module and clinical traits is demonstrated in Fig. 1(g), which showed that SCI had a notable correlation with the green module (correlation = 0.72, p = 0.004). The screening criteria are as follows: GS >0.3, MM >0.8, which was more closely correlated with SCI after screening (r = 0.86, p < 1 × 10^−200^), identified 656 genes [Fig. 1(h)]. A Venn plot was constructed to depict the intersection between the genes of the core module, SCI_DEGs, and NP_DEGs [Fig. 1(i)]. This led us to identify key SCI-related NP genes: Itgb2, Cd52, Ccr5, Fcgr3, Adora3, and Fam83d.

**FIG. 2.**
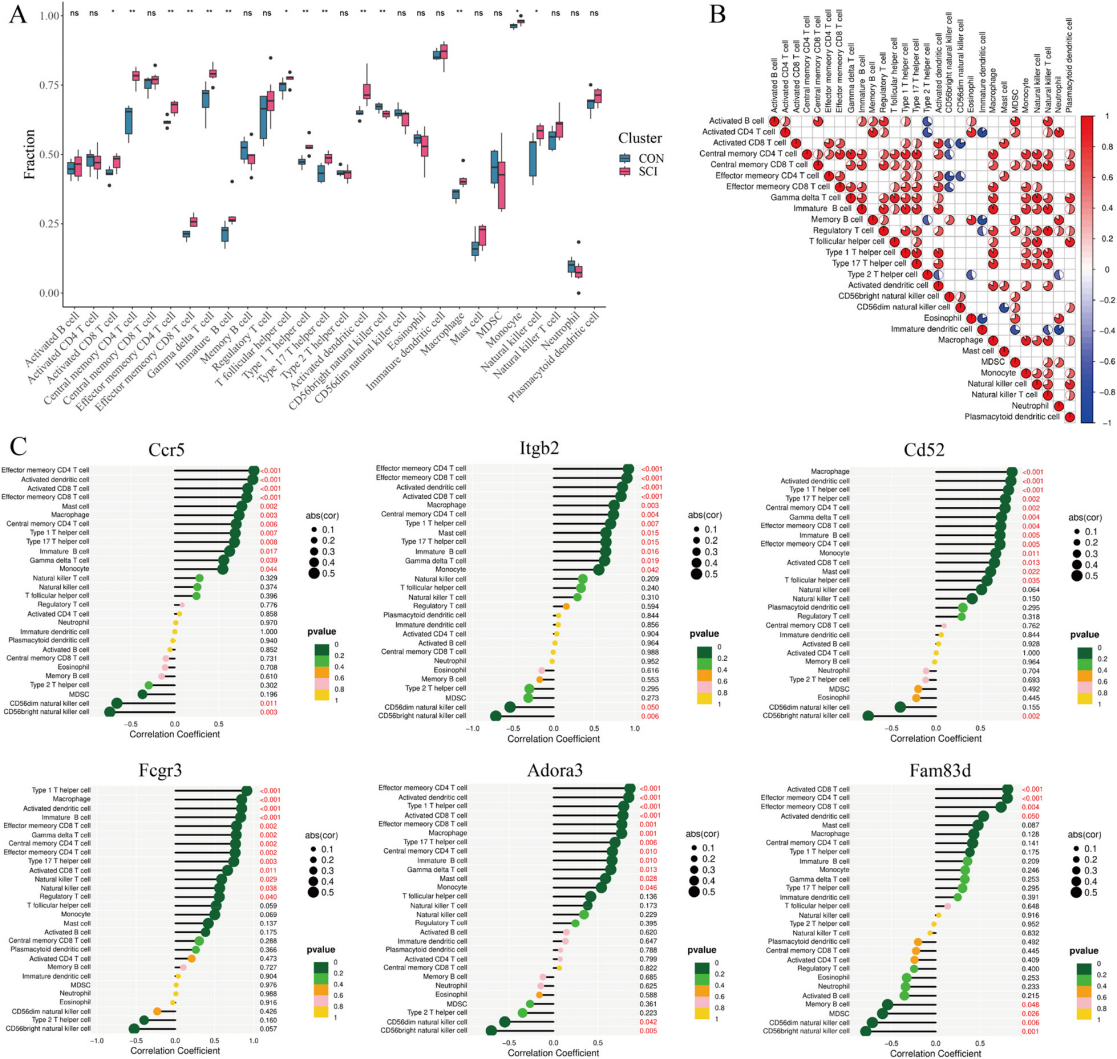
Immune infiltration landscape of spinal cord tissue and immune correlation of hub gene after SCI. (a) Box graph, the difference of immune cell infiltration between Con and SCI groups (symbols ^*^, ^**^, and ns represented p-value <0.05, p-value <0.01, and no statistical significance, respectively). (b) Pie graph, displaying the relationship among 28 types of immune cells. The darker the red, the more significant the positive correlation, and the darker the blue, the more significant the negative correlation. (c) Lollipop chart, the correlation between the hub gene and invasive immune cells. The size of the circle corresponds to the strength of the correlation. p < 0.05 was considered statistically significant.

To further reveal the immune microenvironment in spinal tissues after SCI, the ssGSEA algorithm was used to analyze the specific immune cell types infiltrated into spinal tissues. The boxplot showed differences in immune cell infiltration in DEGs tissues [Fig. 2(a)]. Compared with the CON group, the infiltration levels of T-cell subtypes, macrophages, NK cells, etc., were higher in the SCI group. Additionally, correlation heatmaps showed the relationship between 28 immune cells [Fig. 2(b)], indicating that macrophages were positively correlated with T cells and activated dendritic cells. Subsequently, Spearman correlation analysis was performed to further verify the relationship between key pain-related genes after SCI and immune cell infiltration. The results showed significant correlations between the hub genes and T cells, macrophages, and NK cells [Fig. 2(c)]. Notably, the correlations between Itgb2, Ccr5, Cd52, Fcgr3, Adora3, and macrophages were particularly significant (supplementary material Fig. 1).

### C. scRNA-seq data revealed the heterogeneity of immune cells in spinal cord tissue after SCI

**FIG. 3.**
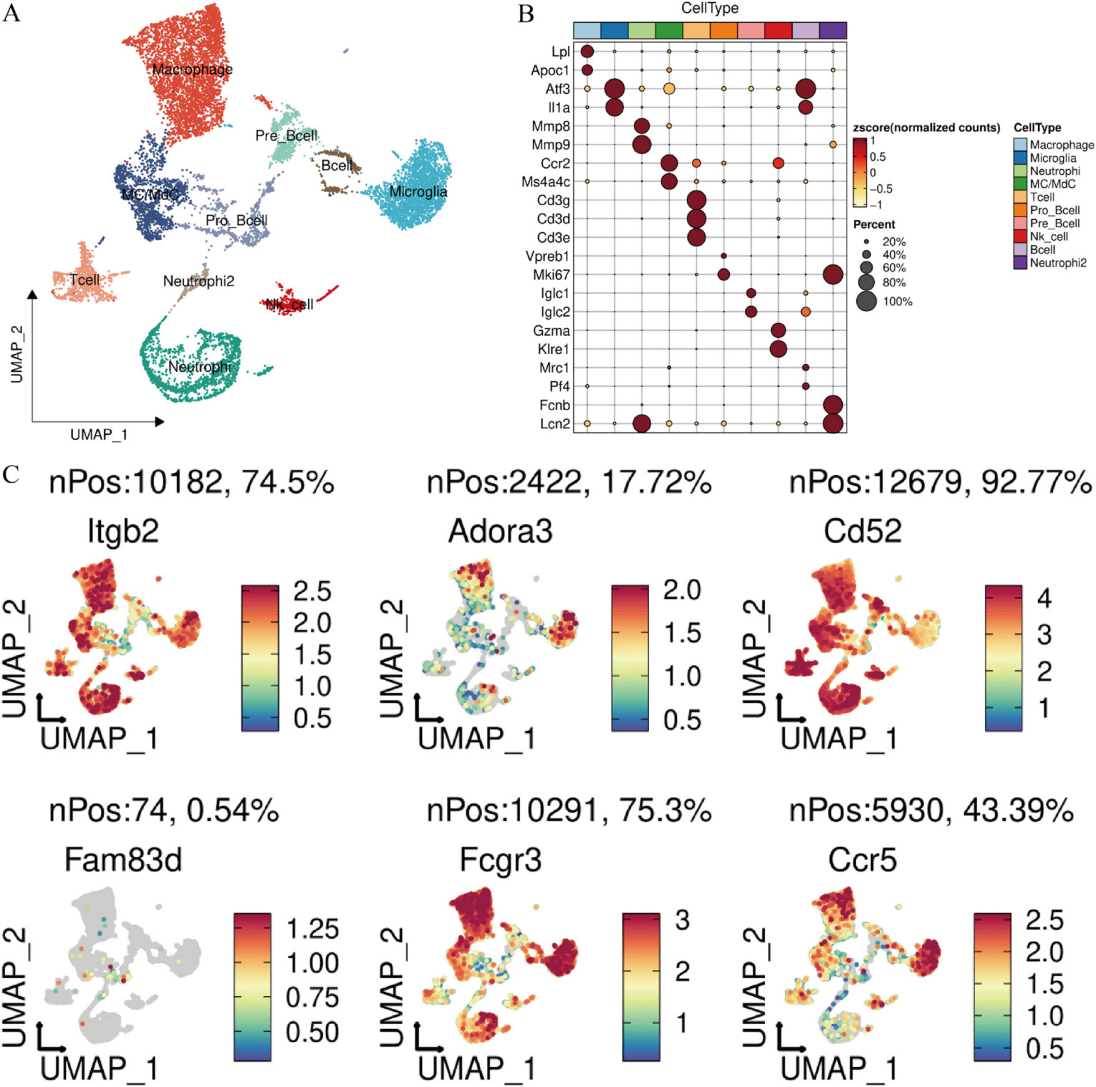
Single-cell data flow analysis and expression profile of the hub gene at the single-cell level. (a) UMAP plot, immune cell subsets annotation in spinal cord tissue of the SCI model. (b) Bubble diagram, annotation of marker genes with subpopulations. (c) UMAP plot, the expression pattern of hub gene at the single-cell level.

Quality control of the gene expression matrix was performed, followed by normalization of the RNA-seq data. Fifteen principal components (PCs) were selected for subsequent analysis (supplementary material Fig. 2). As shown in Fig. 3(a), the immune cells in spinal cord tissue were grouped into ten clusters: macrophages, microglia, NK cells, pre-B cells, pro-B cells, B cells, MC/MdCs, T cells, neutrophils, and neutrophil2. Marker genes for each subpopulation were identified [Fig. 3(b)]. The expression patterns of the hub genes were examined at the single-cell level, and significant expression was found in macrophages, except for Fam83d [Fig. 3(c)].

### D. Spinal cord tissue cell communication after SCI

**FIG. 4.**
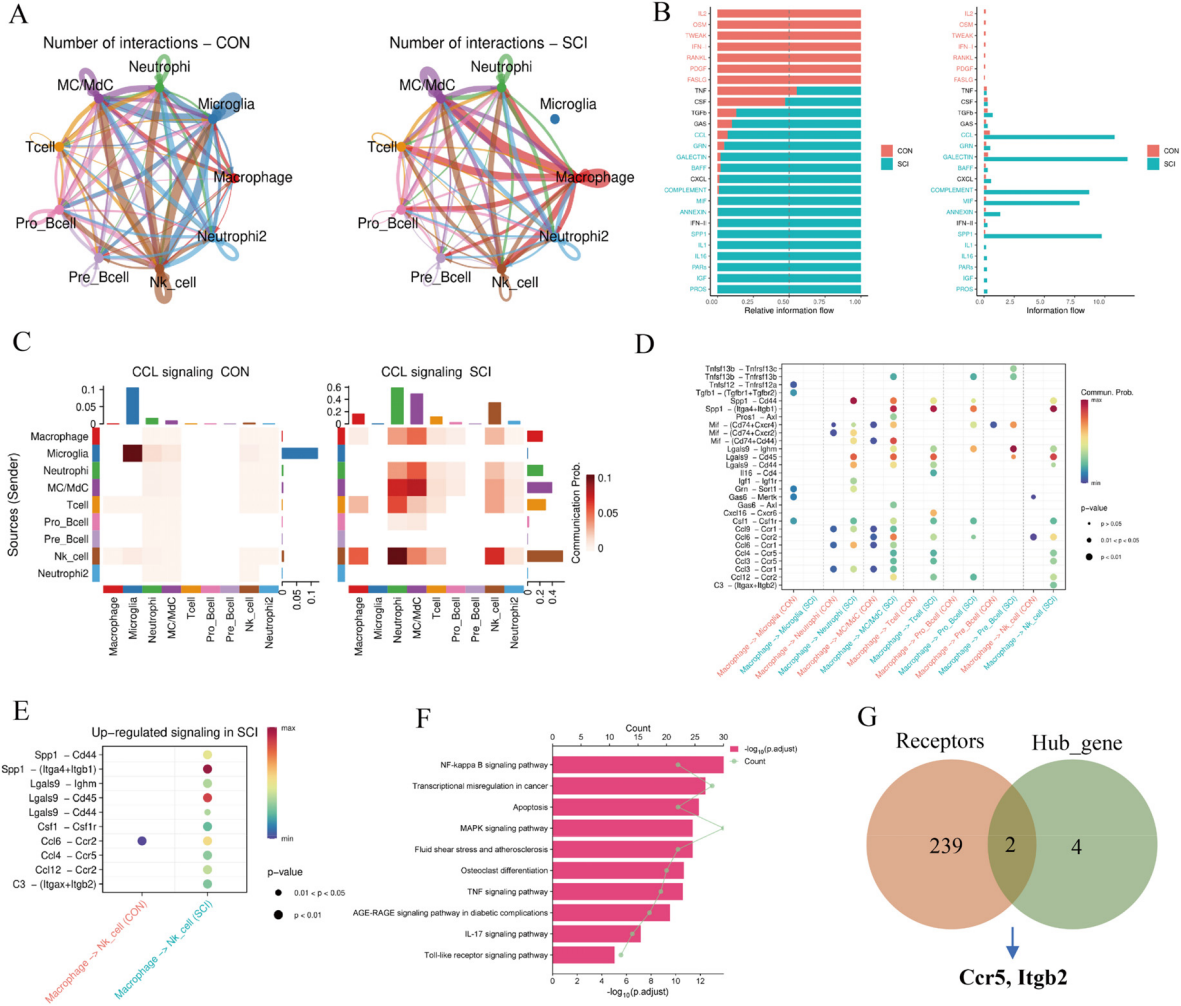
Analysis of cell communication in spinal cord tissue after spinal cord injury. (a) Circle plot displaying the number of communications between any two cell types. (b) Bar chart showing the differences between the control (Con) and SCI groups in overall information flow within the network, ranking important signaling pathways. Red pathways are enriched in the Con group, while green pathways are enriched in the SCI group. (c) Heatmap illustrating the contribution of the CCL signal in different groupings. (d) Bubble plots showing the upregulation (increase) and downregulation (decrease) of signal ligand–receptor pairs in the SCI dataset compared to the Con dataset. (e) Bubble plots showing the upregulated signaling in SCI compared to the Con dataset. (f) Enrichment analysis of target genes in recipient cells, delineating potential functional changes. (g) Venn diagram showing the intersection of genes between receptors and hub genes.

The study revealed strong signal interaction in macrophages, NK cells, MC/MdCs, and neutrophils [Fig. 4(a)]. In comparing the strength of each flow/interaction, the SCI group primarily exhibited signaling through CCL, GALECTIN, COMPLEMENT, MIF, and SPP1 pathways [Fig. 4(b)]. CCL signaling was found to play a critical role in macrophages and NK cells in the SCI group [Fig. 4(c)]. Notably, NK cells exhibited numerous ligand–receptor changes in the SCI group, such as Ccl4–Ccr5 and C3–(Itgax + Itgb2) [Figs. 4(d) and 4(e)].

To further explore macrophage–NK cell communication and downstream signaling changes in NK cells, NicheNet analysis was performed. This analysis identified top-predicted target genes regulated by high-ranking ligands and their corresponding receptors (supplementary material Fig. 3A and B). Pathway analysis of target genes revealed significant enrichment in inflammation-related pathways, including NF-kappa B signaling, apoptosis, MAPK signaling, and TNF signaling [Fig. 4(f)]. By intersecting potential receptors with hub genes, Ccr5 and Itgb2 were identified as key genes mediating macrophage–NK cell interactions after SCI [Fig. 4(g)]. These genes were strongly associated with pain occurrence and are likely critical in the signaling cascade of ligand–receptor interactions and subsequent cellular responses.

### E. Molecular docking studies

**FIG. 5.**
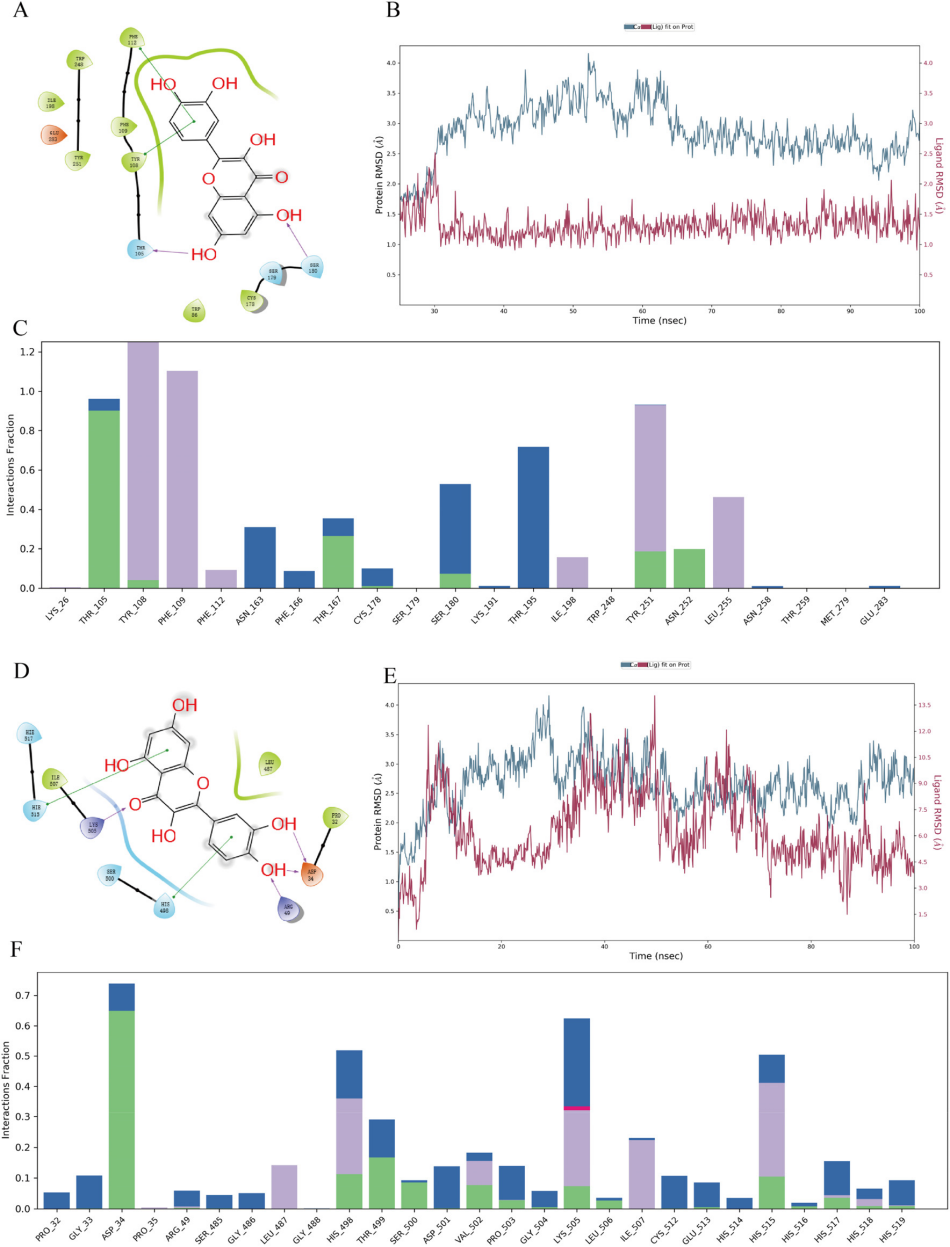
Molecular docking and 100 ns molecular dynamics simulation analysis of the quercetin–Ccr5 and quercetin–Itgb2 complex. (a) 2D ligand–protein interactions of quercetin with Ccr5. (b) RMSD plots of protein and ligand atoms in the quercetin–Ccr5 complex. (c) Histogram showing the fractional interactions of the quercetin–Ccr5 complex. (d) 2D ligand–protein interactions of quercetin with Itgb2. (e) RMSD plots of protein and ligand atoms in the quercetin–Itgb2 complex. (f) Histogram showing the fractional interactions of quercetin–Itgb2 complex.

The two-dimensional (2D) and three-dimensional (3D) structures of quercetin are depicted in supplementary material Fig. 4A. In Fig. 5(a), the solid green lines represent π–π stacking interactions between the ligand and acceptor, and the solid purple lines indicate hydrogen bond interactions. The aromatic ring of quercetin forms π–π stacking interactions with TYR-108 and PHE-112, and its hydroxyl group forms hydrogen bonds with THR-105 and SER-180. supplementary material Fig. 4B depicts the 3D ligand–protein interaction of the quercetin–Ccr5 complex, with purple amino acid residues forming the binding pocket for quercetin in the Ccr5 protein.

Similarly, Fig. 5(d) illustrates the interactions in the quercetin–Itgb2 complex.

The aromatic ring of quercetin forms π–π stacking interactions with HIE-515 and HIE-498, while its hydroxyl group forms hydrogen bonds with ARG-49 and ASP-34. Additionally, the hydrogen on the carboxyl group interacts with LYS-515. supplementary material Fig. 4C shows the 3D ligand–protein interaction of the quercetin–Itgb2 complex, with gray amino acid residues forming the binding pocket for quercetin in the Itgb2 protein. These findings highlight the specific molecular interactions of quercetin with Ccr5 and Itgb2, supporting its potential as a targeted therapeutic agent.

### F. Molecular dynamics simulations

In this study, 100 ns molecular dynamics simulations were conducted for the quercetin–Ccr5 and quercetin–Itgb2 complexes. For the quercetin–Ccr5 complex, supplementary material Fig. 4D illustrates the 2D key ligand–protein interactions and their percentage simulation times. The aromatic ring of quercetin formed π–π stacking interactions with TYR-251 (32%) and TYR-108 (40%, 21%, and 26% for three aromatic rings). Hydroxyl groups on the aromatic ring formed hydrogen bonds with THR-105 (90%) and THR-167 (26%), while THR-195 formed hydrogen bonds via water bridges (61%). According to Fig. 5(b), the average RMSD of protein Cα was 2.64 Å, the ligand atoms were 2.42 Å, and the ligand deviation from its original position was 0.81 Å, indicating low structural deviation. The ligand RMSD stabilized at 2.5 Å in the second half of the simulation, suggesting high stability of the ligand–protein complex. Figure 5(c) shows fractional interaction histograms, with THR-105, TYR-108, PHE-109, and TYR-251 exhibiting the most frequent interactions.

For the quercetin–Itgb2 complex, supplementary material Fig. 4E shows the 2D key ligand–protein interactions. The aromatic ring of quercetin formed π–π stacking interactions with HIS-515 (20%) and HIS-498 (21%), while the carbonyl group formed π–ion interactions with LYS-505 (17%). The hydroxyl group on the aromatic ring formed hydrogen bonds with THR-499 (16%), and ASP-34 formed hydrogen bonds with two aromatic rings (32%). According to Fig. 5(e), the average RMSD of protein Cα was 2.67 Å, while ligand atoms showed a higher RMSD of 6.25 Å, indicating greater deviation from the original position. The ligand RMSD stabilized at 5 Å in the second half of the simulation, suggesting lower stability of the ligand–protein complex. Figure 5(f) shows fractional interaction histograms, with ASP-34, LYS-505, and HIS-515 showing the most frequent interactions. In all, molecular dynamics simulations found that quercetin binding to Ccr5 is more stable, making it a better choice for drug targeting. TYR-108 and PHE-109 are critical amino acids in the quercetin–Ccr5 complex, providing specific and stable amino acid sites for quercetin-targeted gene therapy, which holds significant implications.

### G. In vivo experiments verified the expression changes of hub genes

**FIG. 6.**
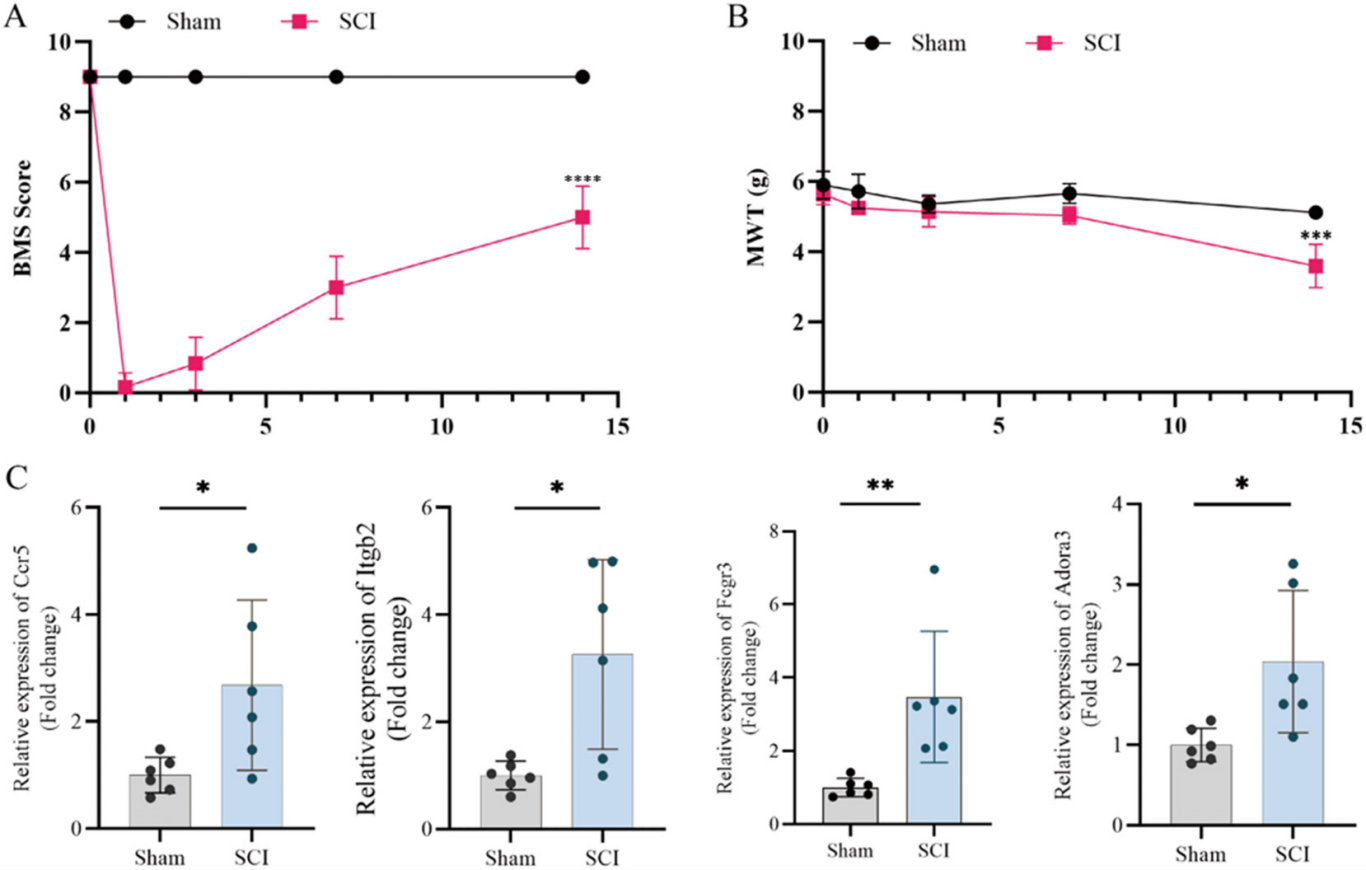
Validation of hub genes in mouse SCI model. (a) and (b) Curves depicting BMS and MWT scores of mice before SCI and at 1, 3, 7, and 14 days after SCI. (c) mRNA expression of hub gene in spinal cord at 14 days after SCI (symbols *, ** and *** represented p-value <0.05, p-value <0.01, and p-value <0.001, respectively).

SCI mouse models were used for *in vivo* validation of the hub gene. As shown in Fig. 6(a), the Basso mouse scale (BMS) score gradually increased starting 3 days post-surgery, with a significant improvement observed at 14 days. Meanwhile, the mechanical withdrawal threshold (MWT) score in the SCI group was significantly lower than that of the Sham group at 14 days, indicating pain development [Fig. 6(b)]. RT-qPCR results revealed significantly increased expression levels of Ccr5, Itgb2, Fcgr3, and Adora3, while Cd52 and Fam83d showed no statistical significance [Fig. 6(c)]. These findings suggest that Ccr5, Itgb2, Fcgr3, and Adora3 may play key roles in pain development following SCI.

### H. Ccr5 inhibitors and quercetin alleviated apoptosis and alleviated pain in SCI models

**FIG. 7.**
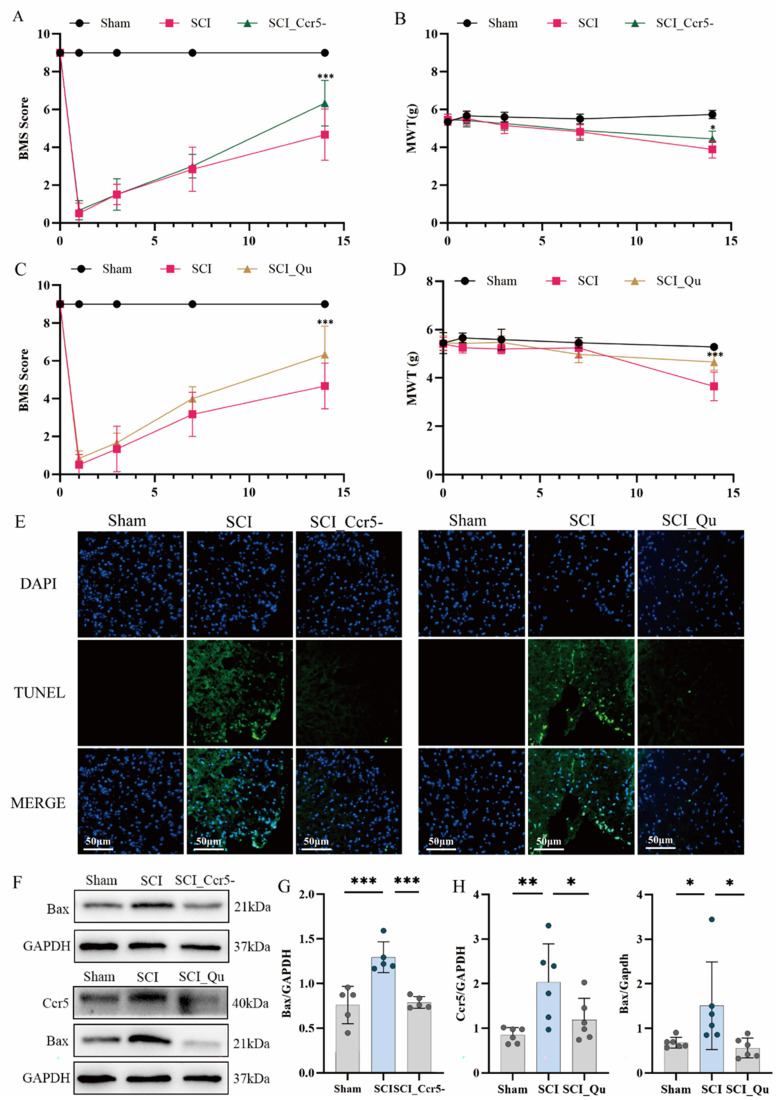
Ccr5 inhibitors and quercetin alleviated the apoptosis of spinal cord tissue and alleviated pain in the mouse SCI model. (a) and (b) Curves showing BMS and MWT scores of mice before SCI and at 1, 3, 7, and 14 days after intraperitoneal injection of a Ccr5 inhibitor. (c) and (d) Curves showing BMS and MWT scores of mice before SCI and at 1, 3, 7, and 14 days after intraperitoneal injection of quercetin. (e) TUNEL staining showed that intraperitoneal injection of Ccr5 inhibitors or quercetin alleviated apoptosis in spinal cord tissue. (f) The expression level of the apoptosis index Bax decreased after treatment with the Ccr5 inhibitor. After quercetin treatment, the expression levels of Ccr5 and Bax decreased. (g) Histogram showing the statistical results of Bax protein expression after Ccr5 inhibitor treatment. (h) Histogram showing the statistical results of Bax protein expression after quercetin treatment (symbols ^*^, ^**^, and ^***^ represent p values <0.05, p values <0.01, and p values <0.001, respectively, compared with the SCI group).

To investigate the role of Ccr5 in NP progression after SCI, intraperitoneal injections of Ccr5 inhibitors were administered in SCI mouse models. As shown in Fig. 7(a), the Ccr5 inhibitor group significantly improved BMS scores and alleviated pain [Fig. 7(b)]. The quercetin group demonstrated even greater improvements in BMS scores and pain thresholds at 2 weeks post-SCI compared to the Ccr5 inhibitor group [Figs. 7(c) and 7(d)]. The analgesic effects of Ccr5 inhibitors and quercetin are comparable to pregabalin (supplementary material Fig. 5). To further validate Ccr5 as a potential target for Ccl4, we injected Ccl4 recombinant protein intraperitoneally to establish a model of spinal cord injury. The results showed that the Ccl4 recombinant protein aggravated the pain after SCI. However, the pain-promoting effects of Ccl4 can be reversed by Ccr5 inhibitors (supplementary material Fig. 6). Apoptosis in the spinal cord of SCI mice was assessed using terminal deoxynucleotidyl transferase dUTP nick end labeling (TUNEL) staining and western blot analysis. TUNEL staining showed that both Ccr5 inhibitors and quercetin reduced spinal cord apoptosis [Fig. 7(e)]. Western blot analysis further confirmed that both treatments suppressed the upregulation of the apoptosis marker Bax [Figs. 7(f)–7(h)], with statistically significant differences.

## III. DISCUSSION

The mechanisms underlying NP after SCI have not yet been clarified, and there is a lack of effective treatments. This underscores the urgent need to explore the relationship between SCI and NP and to develop therapeutic strategies that address this intersecting pathology.^25^ Recent research has focused extensively on the changes in the immune microenvironment following SCI to identify new therapeutic approaches that can prevent or mitigate the development of NP.^26,27^ The study examines the immune infiltration patterns, key biomarkers, and pharmacological strategies in spinal cord tissues post-SCI to offer new insights into the clinical management of comorbidities. By integrating transcriptome-wide data, 1050 DEGs significantly enriched in pathways related to cell interaction and inflammation, such as immune effector processes, cytokine receptor binding, cytokine–cytokine receptor interaction, Th17 cell differentiation, the JAK–STAT signaling pathway, and Th1 and Th2 cell differentiation, were identified.^15,28,29^ These findings indicate significant changes in the spinal cord immune microenvironment following SCI.^30^ Further immune infiltration analysis revealed that various immune cells, including neutrophils, macrophages, B lymphocytes, T lymphocytes, and NK cells, are closely associated with SCI. Combining WGCNA and ssGSEA, NP-related genes implicated in the immune response following SCI: Itgb2, Ccr5, Cd52, Fcgr3, and Adora3. These genes are closely linked to immune response and inflammation processes. Chidlow *et al.* found that VEGF164 differentially regulates neutrophil and T-cell adhesion through ItgaL- and ItgaM-dependent mechanisms.^31^ Adora3, an adenosine receptor, affects pain perception by regulating immune and inflammatory responses;^32^ Fcgr3 is associated with Fc receptor-mediated immune responses;^33^ and Ccr5, a chemokine receptor, is important for immune cell chemotaxis and activation. Raghu *et al.* found^34^ that CCL2/CCR2 mediates monocyte recruitment, inflammation, and cartilage destruction in osteoarthritis. The abnormal expression of these genes may influence neuroinflammation and pain transmission post-SCI through various mechanisms.

Despite various immune cell infiltrations in post-SCI spinal cord tissues, the precise mechanisms of their interactions remain fully delineated.^35^ This study uses single-cell data to further analyze the heterogeneity of immune cells within spinal cord tissues.^36^ We discovered close interactions between macrophages and various immune cells, especially NK cells, in which ligand receptors such as Ccl4–Ccr5 and C3–(Itgax + Itgb2) play an important role. In addition, we further revealed that signals received by NK cells influence the expression of a series of target genes, triggering significant changes in pathways such as NF-kappa B signaling,^37^ apoptosis,^20^ MAPK signaling,^38,39^ and TNF signaling,^40^ all closely linked to neuroinflammation and pain following SCI. It has been reported that SARM1 promotes neuroinflammation and inhibits neural regeneration after spinal cord injury through NF-kappa B signaling.^41^ Activation of this signaling pathway can lead to the recruitment and activation of immune cells and the release of numerous inflammatory cytokines, thereby amplifying neuroinflammatory responses.^42,43^ This neuroinflammation not only directly affects neurons, causing pain but also alters neuronal electrophysiology and synaptic transmission mechanisms, leading to abnormal and amplified pain signaling.^44,45^ Our research suggests that the Ccl4–Ccr5 and C3–(Itgax + Itgb2) signaling pathways might be a critical regulatory axis in the development of NP following SCI. It mediates the interaction between macrophages and NK cells, activates downstream inflammatory pathways, and initiates the apoptosis program.^46^ These findings provide new insights into the mechanisms of cell death after spinal cord injury and provide potential targets for the treatment of Ccr5 and Itgb2.

Quercetin, a natural flavonoid with anti-inflammatory, antioxidant, and anti-apoptotic properties,^47^ has not been fully explored for its potential in treating NP post-SCI. Through molecular docking and molecular dynamics simulations, this study investigates the potential therapeutic strategies involving quercetin. We found that quercetin has significant binding sites with Ccr5 and Itgb2, with particularly stable binding to Ccr5. Critical amino acid residues such as TYR-108 and PHE-109 were identified in the quercetin–Ccr5 complex, providing specific and stable drug targets with important clinical implications. Luo *et al.* research found that quercetin inhibits macrophage pyroptosis through competitive binding of Arg483 to KEAP1.^48^ Experimental studies demonstrated that quercetin or Ccr5 inhibitors improved motor function and alleviated NP post-SCI. Interestingly, the analgesic effects of quercetin and Ccr5 inhibitors were comparable to those of pregabalin. Additionally, quercetin and Ccr5 inhibitors reduced apoptosis in spinal cord tissues post-SCI. We hypothesize that quercetin may interfere with macrophage–NK cell communication by blocking the Ccl4–Ccr5 pathway, reducing immune cell recruitment and activation, and lowering inflammatory cytokine release, thereby mitigating neuroinflammation and apoptosis and alleviating NP.

## IV. CONCLUSION

NP post-SCI is a prevalent and challenging complication that severely impacts patients' quality of life. By integrating single-cell and bulk transcriptome data, the study identified the roles of key pain-related genes (Ccr5, Itgb2, Fcgr3, and Adroa3) and unveiled potential signaling pathways in macrophage and NK cell communication, particularly the Ccl4–Ccr5 pathway. Molecular docking and molecular dynamics simulations demonstrated stable amino acid binding sites for quercetin and Ccr5 at TYR-108 and PHE-109, presenting potential opportunities for further mutation experiments and targeted drug therapy with quercetin. The study found that both Ccr5 inhibitors and quercetin could improve BMS scores post-SCI, increase pain thresholds, and reduce apoptosis. It is hypothesized that quercetin may alleviate NP by inhibiting the Ccl4–Ccr5 signaling pathway, disrupting immune cell communication, and reducing spinal cell apoptosis, offering a promising target for NP treatment after SCI.

## V. METHODS

### A. Data download and preprocessing

Bulk transcriptome datasets (GSE93976 and GSE171441) and single-cell transcriptome datasets (GSE182803) related to SCI and NP bulk transcriptome datasets (GSE111216) were obtained from the Gene Expression Omnibus (GEO) database (https://www.ncbi.nlm.nih.gov/geo/). Data analysis was performed using the R language version 4.1.3 (https://www.r-project.org/). The bulk transcriptome datasets were integrated, and batch effects were corrected using functions from the “limma” package.^49^ For visualization post-batch effect correction, UMAP plots were generated using the “ggplot2” package.^50^

### B. Differentially expressed genes identification and enrichment analysis

Differential gene expression analysis between control (CON) and SCI groups was performed using linear models in the “limma” package. DEGs for NP were identified and filtered based on the criteria of p-value <0.05 and |log_2_ FC| >1. We used the “clusterProfiler,” “org.Mm.eg.db,” “enrichplot,”^51^ and “ggplot2” packages to conduct Gene Ontology (GO)^52^ and Kyoto Encyclopedia of Genes and Genomes (KEGG)^53^ pathway analyses. Additionally, NP-related genes were identified from GSE111216 (excluding duplicate items, p < 0.05, logFC > 0.5), resulting in 152 NP_DEGs.

### C. Weighted gene co-expression network analysis

Weighted gene co-expression network analysis (WGCNA)^54^ was performed to identify key regulatory genes and their correlation with SCI. The top 5000 genes with the highest median absolute deviation were analyzed. Outliers were removed via hierarchical clustering, and a scale-free network was constructed (β = 6, R^2^ = 0.85). Gene modules were generated using dynamic hybrid cutting (minModuleSize = 50, mergeCutHeight = 0.25), and module-SCI correlations were visualized in a heatmap. Genes with gene significance (GS >0.3) and module membership (MM >0.8) were identified. The intersection of genes from relevant modules, SCI_DEGs, and NP_DEGs was defined as key SCI-related pain genes, visualized with a Venn diagram using the “VennDiagram” package.^55^

### D. Immune cell infiltration and correlation analysis

The ssGSEA algorithm was used to evaluate differences in immune cell populations between the CON and SCI groups, with p < 0.05 considered statistically significant. Box plots were generated using the “ggplot2” package for visualization. Correlations among the 28 infiltrating immune cells were illustrated with the “corrplot” package. Furthermore, the correlation between key pain-associated genes and infiltrating immune cells was analyzed and visualized as lollipop charts using the “ggplot2” package.

### E. scRNA-seq data processing and identification of cell types

Single-cell analysis was conducted using the “Seurat”^56,57^ and “SCP” packages. Cells were filtered based on nFeature_RNA >500, nFeature_RNA <4000, and percent.mt <5%. Gene expression levels were normalized using the “LogNormalize” algorithm, and the top 2000 highly variable genes (HVGs) were identified with the “VST” algorithm. PCA was performed to identify significant principal components (PCs), with p-value distributions visualized using the JackStraw and ScoreJackStraw functions. Batch effects were corrected using the “Harmony” package. Cells were clustered into ten groups using the FindClusters function (resolution = 0.12) and manually annotated with CellMarker and panglaoDB. Key gene expression patterns were visualized with UMAP plots.

### F. Cell communication

The “CellChat”^16^ and “NicheNet”^17^ packages were used to analyze cell communication networks and interactions within biological systems. “CellChat” was first employed to identify communication networks and ligand–receptor interactions between different cell types in SCI spinal cord tissue. Subsequently, the “NicheNet” package was utilized to analyze the activation of relevant pathways and the biological effects following the reception of signals by receiver cells.

### G. Molecular docking and molecular dynamics simulation

The Schrödinger 2021 System Builder module was used for molecular docking and molecular dynamics simulations. The two-dimensional (2D) structure of quercetin was obtained from the PubChem database, while protein structures for Ccr5 (PDB: 6AKX) and Itgb2 (PDB: 5E6X) were retrieved from the PDB database. The ligand (quercetin) was energy-minimized and converted to a three-dimensional (3D) conformation, while receptor proteins were prepared by assigning bond orders, adding hydrogens, creating zero-order bonds to metals, and forming disulfide bonds. The open-source software CavityPlus was employed, with the target protein specified by its PDB ID and all other parameters set to their default values. Among the binding pockets identified by the calculations, the pocket with the highest druggability score was selected as the potential binding site for small-molecule compounds. The geometric center of this pocket was used as the central coordinate to define the binding site, which was subsequently established in Schrödinger. The corresponding binding pocket coordinates and surrounding amino acid residues are shown in supplementary material Table 1. For molecular dynamics simulations, the SPC solvent model and an orthorhombic system shape were used with the OPLS4 force field. Na^+^ and Cl^−^ ions were added to maintain electrical neutrality, and the system was set to a 0.15 M NaCl solution. Simulations were conducted using the Desmond module for 100 ns with a step length of 100 ps. The temperature was maintained at 300 K, and the pressure at 1.01 bar, with default settings for other parameters. The 6AKX model contained 47 397 atoms, and the 5E6X model contained 44 021 atoms. Post-simulation analysis was performed using the Simulation Interactions Diagram, generating a report detailing protein–ligand RMSD, ligand RMSF, protein–ligand contacts, and other key results.

### H. Mouse SCI models

Adult male C57BL/6 mice were randomly assigned to six groups: (1) Sham group (n = 8); (2) SCI + phosphate-buffered saline (PBS) group (n = 8); (3) SCI + Ccr5 inhibitor group (n = 8); (4) SCI + quercetin group (n = 8); (5) SCI + Ccl4 recombinant protein (n = 8); and (6) SCI + Ccl4 recombinant protein + Ccr5 inhibitor (n = 8). Mice were anesthetized with Avertin, and SCI models were created by exposing the T9–T10 spinal cord segments after laminectomy at the 10th thoracic vertebra level and then lightly striking the T9 segments with an SCI hammer (parameters: injury velocity of 1.5 m/s, injury depth of 0.2 mm, residence time of 0.5 s, and percussion head diameter of 1.3 mm). Following surgery, muscles and skin were sutured, the bladder was manually expressed twice daily for 2 weeks, and ceftriaxone (16 mg/kg) was administered intraperitoneally twice daily for 7 days. In the Ccr5 inhibitor group, 15 *μ*g/10 g decitabine (HY-14231, MCE, USA) was injected intraperitoneally once a day for 14 days. In the quercetin group, mice were injected intraperitoneally with 15 *μ*g/10 g quercetin (HY-18085, MCE, USA) once a day for 14 days. Motor function was evaluated using the BMS before surgery and on days 1, 3, 7, and 14 after surgery. MWT was measured using von Frey filaments before surgery and on days 1, 3, 7, and 14 after surgery.

### I. Real-time quantitative polymerase chain reaction (qPCR)

Total RNA was extracted using Trizol reagent (BS258A; Beyotime, China), and reverse transcription was performed using the RevertAid RT Reverse Transcription Kit (R222-01, Vazyme Biotech, China), following the manufacturer's guidelines. qPCR amplification was executed using the Cham Q Universal SYBR qPCR Master Mix kit (Q711-02, Vazyme Biotech, China). The amplification protocol included an initial phase at 95 °C for 1 min, followed by 40 cycles at 95 °C for 15 s, and 50 °C for 30 s. Primer sequences are detailed in Table III.

**TABLE III.** Primers used for real-time quantitative PCR.

Gene name	Forward (5′–3′)	Forward (5′–3′)
GAPDH	TGGGTGTGAACCATGAGAAGT	AGACAATGAAATAGACGGTGGTG
Adora3	ACGGACTGGCTGAACATCAC	AGACAATGAAATAGACGGTGGTG
Ccr5	ATGGATTTTCAAGGGTCAGTTCC	CTGAGCCGCAATTTGTTTCAC
Cd52	ATCCTTGGGACAAGCCACTAC	GGCACATTAAGGTATTGGCAAAG
Itgb2	CAGGAATGCACCAAGTACAAAGT	GTCACAGCGCAAGGAGTCA
Fam83d	CGAACGAGTCCTCCGCTAC	TGGACATTCAAGTCAGTTACCGAA
Fcgr3	AATGCACACTCTGGAAGCCAA	CACTCTGCCTGTCTGCAAAAG

### J. Western blotting

Two weeks after establishing the SCI model, spinal cord tissues were collected 1 cm above and below the T9 segment. These specimens were frozen in liquid nitrogen or fixed in 4% paraformaldehyde overnight. The tissues were then disrupted in RIPA buffer containing a protease inhibitor (CW2333S, Cowin Biotech, China) and a phosphatase inhibitor (CW2200S, Cowin Biotech, China). Protein concentration was quantified using the BCA assay (23225, Thermo Fisher Scientific, USA). Electrophoresis was performed on a 12% SDS–PAGE gel, and proteins were transferred to a PVDF membrane. The membrane was blocked at room temperature with 5% skim milk for 2 h, followed by incubation with primary antibodies, including GAPDH (1:1000, CST, Cat# 18665), Ccr5 (1:1000, CST, Cat# 18665), and Bax (1:2000, ab128878), overnight at 4 °C. After washing with TBST (three cycles of 5 min each), the membrane was incubated with goat anti-rabbit IgG (1:5000; ab205718, Abcam) and goat anti-mouse IgG (1:5000; ab150113, Abcam) at room temperature for 1 h. Protein detection was performed using Biodlight™ ECL Chemiluminescent HRP Substrate (High Sensitivity) (BLH01S100, Bio World, USA) and the TANON-5200 chemiluminescence imaging system (Tanon Technology, Beijing, China). ImageJ 6.0 software was used for result analysis.

### K. Terminal deoxynucleotidyl transferase dUTP nick end labeling (TUNEL) staining

According to the protocol of the One-step TUNEL In Situ Apoptosis Kit (Green, PriFluor 488) (P-CA-302, Procell, China). TUNEL staining was performed to detect apoptotic cells. Tissue sections (10 *μ*m) were prepared and mounted on slides, followed by fixation with 4% paraformaldehyde. Permeabilization was achieved using 0.1% Triton X-100 in PBS. The TUNEL reaction mixture (Green, PriFluor 488) was applied and incubated at 37 °C for 60 min. Post-incubation, slides were washed with PBS, optionally counterstained with DAPI, and mounted with antifade medium. Apoptotic cells were identified under a fluorescence microscope by their green fluorescence.

### L. Statistical analysis

All statistical analyses were conducted using R software (version 4.1.3). Comparisons between two groups were assessed using the independent sample t-test, while differences among multiple groups were evaluated using one-way analysis of variance (ANOVA). Additional statistical calculations were performed using Prism 9.0 and ImageJ software. Figures were created using Adobe Illustrator software. p < 0.05 was considered statistically significant.

## SUPPLEMENTARY MATERIAL

See the supplementary material for detailed experimental protocols, as well as supporting figures and tables that further validate the findings presented in the main text.

## Data Availability

The data that support the findings of this study are openly available in NCBI at https://www.ncbi.nlm.nih.gov/geo/query/acc.cgi?acc=GSE93976, Ref. 58; NCBI at https://www.ncbi.nlm.nih.gov/geo/query/acc.cgi?acc=GSE171441, Ref. 59; NCBI at https://www.ncbi.nlm.nih.gov/geo/query/acc.cgi?acc=GSE182803, Ref. 60; and NCBI at https://www.ncbi.nlm.nih.gov/geo/query/acc.cgi?acc=GSE111216, Ref. 61.
